# Silenced lncRNA DDX11-AS1 or up-regulated microRNA-34a-3p inhibits malignant phenotypes of hepatocellular carcinoma cells via suppression of TRAF5

**DOI:** 10.1186/s12935-021-01847-6

**Published:** 2021-03-22

**Authors:** Gangqiang Ding, Yanli Zeng, Dongqiang Yang, Can Zhang, Chongshan Mao, Erhui Xiao, Yi Kang, Jia Shang

**Affiliations:** Department of Infectious Diseases, Henan Key Laboratory for Liver Disease, Henan Provincial People’s Hospital, People’s Hospital of Zhengzhou University, No. 7 Weiwu Road, Zhengzhou, 450003 Henan China

**Keywords:** Hepatocellular carcinoma, Long noncoding RNA DDX11-AS1, MicroRNA-34a-3p, Tumor necrosis factor receptor-associated factor 5, Proliferation, Metastasis

## Abstract

**Background:**

Studies have discussed long noncoding RNA DDX11-AS1 (DDX11-AS1)-mediated downstream mechanism in hepatocellular carcinoma (HCC). The goal of this study was to investigate the regulatory mechanism of DDX11-AS1-mediated microRNA-34a-3p (miR-34a-3p)/tumor necrosis factor receptor-associated factor 5 (TRAF5) axis on HCC cells.

**Methods:**

DDX11-AS1, miR-34a-3p and TRAF5 expression levels in HCC were detected. The correlation of DDX11-AS1, miR-34a-3p and TRAF5 in HCC patients was analyzed by Pearson test. HCC cells were transfected with corresponding plasmid/oligonucleotide, and cell proliferation, migration, invasion, apoptosis and tumor formation ability were detected. Bioinformatics software, dual luciferase report experiment and RNA-pull down experiment analysis were applied to verify the targeting relationship between DDX11-AS1, miR-34a-3p and TRAF5.

**Results:**

Elevated DDX11-AS1 and TRAF5 and reduced miR-34a-3p exhibited in HCC. Silenced DDX11-AS1 or up-regulated miR-34a-3p inhibited the proliferation, migration, invasion, promoted apoptosis of HCC cells and repressed the tumor growth in nude mice. In addition, DDX11-AS1 bound to miR-34a-3p to target TRAF5. Silencing TRAF5 or elevating miR-34a-3p expression mitigated up-regulated DDX11-AS1-mediated promotion of tumor growth.

**Conclusion:**

Silenced DDX11-AS1 or up-regulated miR-34a-3p inhibits HCC cell growth via elevation of TRAF5, which could be of great benefit to find early diagnostic markers for HCC patients.

**Supplementary Information:**

The online version contains supplementary material available at 10.1186/s12935-021-01847-6.

## Background

Hepatocellular carcinoma (HCC) is an exigent health problem around the world nowadays because of its high occurrence and lethality, and late discovery [1]. HCC is also found as the main kind of primary liver cancer [2]. The higher morbidity and mortality of HCC in Asian-Pacific areas result from the prevalence of hepatitis B and C virus infection [3]. The multimodal therapies of HCC have been developed, such as surgical resection, liver transplantation, radiofrequency ablation/percutaneous ethanol injection [4]. The prognosis of HCC patients varies and largely depends on the tumor burden score [5]. Therefore, it is vital to find new treatment targets based on illuminating the potential mechanism related to HCC occurrence and development.

The molecular functions of long noncoding RNA (lncRNA) consist of the regulation of transcript stability, translation, transcription, and cell signaling together with nuclear scaffolding [6]. LncRNA DDX11 antisense RNA 1 (DDX11-AS1), also named as cohesion regulator noncoding RNA, is reported to be key in a variety of carcinomas, such as esophageal cancer, HCC and bladder cancer [7–9]. There is a study indicating that DDX11-AS1 may be a new oncogene in hepatocarcinogenesis, which offers a possible therapeutic target for HCC therapy [10, 11]. Moreover, Gene Set Enrichment Analysis on DDX11-AS1 expression has suggested that DDX11-AS1 influences the gene expression connected with HCC cell cycle, proliferation and differentiation, exhibiting a necessary role of DDX11-AS1 in the formation of HCC [12]. MicroRNAs (miRNAs), small non-coding RNAs, are implicated in post-transcriptional modulation of gene expression [13]. Previous researches have reported that there is a correlation between miR-34a with HCC. For example, a study has manifested that miR-34a is a possible onco-suppressor for HCC [14]. In the meantime, miR-34a also could be regarded as a suppressor of lots of cancers including HCC, ovarian cancer and prostate cancer [15–17]. A study has revealed that up-regulated miR-34 in hepatic stellate cells relieves the progression of liver fibrosis [18]. In human cancers, genetic changes in the gain and loss of function of different tumor necrosis factor receptor-associated factors (TRAFs) are common [19]. TRAF6 is the abundantly expressed gene that relates to tumorigenesis and development [20]. In fact, TRAF6 is the regulatory actor in liver cancer, as to cell apoptosis [21]. TRAF5, is also a member of the recently identified TRAF family [22] that is clearly up-regulated in HCC [23]. What’s more, TRAF5 silence could suppress HCC progression [24]. Thus, we aim to investigate the regulatory mechanism of DDX11-AS1/miR-34a-3p/TRAF5 signaling axis on HCC cells.

## Materials and methods

### Compliance with ethical standards

The study was permitted by the Institutional Review Board of Henan Key Laboratory for Liver Disease, Henan Provincial People’s Hospital; People’s Hospital of Zhengzhou University and followed the tenets of the *Declaration of Helsinki*. All participants signed a document of informed consent. Animals were treated humanely and the protocol was approved by the Institutional Animal Care and Use Committee of Henan Key Laboratory for Liver Disease, Henan Provincial People’s Hospital; People’s Hospital of Zhengzhou University.

### Study subjects

From January 2016 to March 2019, 91 cases of fresh HCC tissues (cancerous tissues and adjacent tissues) were collected, all of which were pathologically diagnosed as HCC. The tissue samples were placed in the freezer tube within 30 min and immediately frozen at -80℃. The patients did not receive radiotherapy or chemotherapy before surgery [10].

### Cell culture

Human normal hepatocyte LO2 and HCC cell lines SMMC-7721 and SK-hep1 were purchased from American Type Culture Collection (VA, USA) and cultured in Dulbecco’s Modified Eagle Medium (DMEM) (Invitrogen, Carlsbad, California, USA) containing 10% fetal bovine serum (FBS) (Invitrogen), 100 IU/mL penicillin and 100 mg/mL streptomycin. When the cell confluence reached about 90%, the cells were trypsinized and passaged.

### Cell transfection

SMMC-7721 and SK-hep1 cells were transfected with DDX11-AS1 low-expression vector negative control (NC), DDX11-AS1 low-expression vector, miR-34a-3p mimic NC, miR-34a-3p mimic, DDX11-AS1 overexpression vector + miR-34a-3p mimic NC, DDX11-AS1 overexpression vector + miR-34a-3p mimic, DDX11-AS1 overexpression vector + silenced TRAF5 NC, or DDX11-AS1 overexpression vector + silenced TRAF5.

The lentivirus containing sh-DDX11-AS1 and sh-NC, miR-34a-3p mimic and its NC were purchased from GenePharma Co. Ltd. (Shanghai, China). pcDDX11-AS1 was constructed by RiboBio Co., Ltd. (Guangdong, China). The cells were seeded into 12-well plates 24 h before transfection, and each well was added with 1.5 mL of penicillin/streptomycin-free complete culture medium. When the cell confluence reached about 60%, SMMC-7721 and SK-hep1 cells were stably transfected in lipofectamine 2000 (Invitrogen) for 6 h. The medium was changed, and the cells were collected for subsequent experiments after 48-h culture.

### Cell proliferation assay

The cells were seeded and 4 duplicated wells were set. After culture for 24, 48 and 72 h, Cell counting kit (CCK)-8 reagent (Beyotime Biotechnology Co., Ltd., Shanghai, China) was used to detect the optical density value at 450 nm.

### Colony formation assay

Cells were trypsinized and 200 cells were seeded into a 6-well plate for 2–3 w. Microscopic cell colonies were fixed with 4% paraformaldehyde, stained with Giemsa application solution for 60 min and counted under a microscope [25].

### Cell apoptosis assay

The cells were centrifuged at 1000 r/min for 5 min, and resuspended by adding 400 μL Binding Buffer (1 × 10^6^ cells/mL). Then the cells were reacted with 5 μL Annexin V-fluorescein isothiocyanate (FITC) and 5 μL propidium iodide (PI) for 15 min. Apoptosis was detected via a flow cytometer (BD FACS Arial I cell sorter).

### Cell migration assay

The cells were seeded and cultured for 24 h, and then were scratched with a sterile pipette evenly. The cells and fragments were washed with phosphate buffer saline (PBS) and then the medium was replaced with serum-free medium. The scratch widths at 0 h and 24th h were recorded under the microscope, and the migration rate was calculated.

### Cell invasion assay

The cell invasion ability was evaluated using Transwell Chambers (Corning Glass Works, Corning, NY, USA). The cells were resuspended firstly with serum-free DMEM. The cells (1 × 10^5^) were added into the upper chambers with matrigel (Corning) in the bottom of chambers. Then the cells were added in 24-well plates with DMEM containing 10% FBS. After 24 h, the cells in the chamber were removed, while the cells outside the Transwell chamber were fixed and stained with crystal violet solution, and counted under an inverted microscope.

### Reverse transcription quantitative polymerase chain reaction (RT-qPCR)

The total RNA was extracted with Trizol (Invitrogen). The reverse transcription mixture kit (GoScript M, Reverse Transcription Mix, Random Primers) (Promega, Madison, WI, USA) was used for the reverse transcription of RNA into cDNA. RNA from cell lines was extracted after direct lysis and reversely transcribed to cDNA using the same method. Primers and loading control glyceraldehyde phosphate dehydrogenase (GAPDH) were synthesized by Sangon (Shanghai, China) (Table 1). PCR amplification was performed. The relative expression was calculated by 2^−△△Ct^ method.

**Table 1:** Primer sequence

Genes	Primers (5′→3′)
miR-34a-3p	Forward: CGGGATCCGCAGCCTCTCCATCTTC
	Reverse: GGAATTCGGCTAGGAGGATCAACACAC
U6	Forward: CGCAAGGATGACACGCAAATTCG
	Reverse: CAGTGCAGGGTCCGAGGT
DDX11-AS1	Forward: CTGGCTACTCTTCCTCCTGG
	Reverse: CAGAGGACATGTGGGAGGTT
TRAF5	Forward: CCTACGGAAAGACCTGAAAGAGC
	Reverse: GGGTATTCAGGACACAAGTTTTCC
Ki67	Forward: GAGGAGAAACGCCAACCAAGAG
	Reverse: TTTGTCCTCGGTGGCGTTATCC
Caspase-3	Forward: GGAGTCTGACTGGAAAGCCGAA
	Reverse: CTTCTGGCAAGCCATCTCCTCA
GAPDH	Forward: TGCACCACCAACTGCTTAGC
	Reverse: GGCATGGACTGTGGTCATGAG

MiR-34a-3p, MicroRNA-34a-3p; DDX11-AS1, DDX11 antisense RNA 1; TRAF5, tumor necrosis factor receptor-associated factor 5; GAPDH, glyceraldehyde-3-phosphate dehydrogenase

### Western blot analysis

The total proteins from tissues and cells were extracted and implemented electrophoresis. The target protein was transferred to membranes and blocked with 20 mL of blocking solution for 1 h. Then the protein was incubated with primary antibodies GAPDH and TRAF5 (1: 1000, Cell Signaling Technology, Danvers, MA, USA) and secondary antibody for 1 h. The membrane was developed by enhanced chemiluminescence solution for 1 min. Imaging was performed, and protein gray values were analyzed.

### Dual luciferase reporter gene assay

Through bioinformatics website https://cm.jefferson.edu/rna22/Precomputed/, the binding sites of DDX11-AS1 and miR-34a-3p were predicted. The binding sites of DDX11-AS1 and miR-34a-3p were cloned into the pGL3 plasmid, or mutated and then cloned into the pGL3 plasmid to obtain pGL3-DDX11-AS1 wild type (WT) or mutant type (MUT) vector. The cells were seeded into 24-well plates to adhere to the wall and co-transfected with vectors of pGL3-DDX11-AS1 and miR-34a-3p mimic via lipofectamine 3000 (ThermoFisher Scientific, MA, USA). After 48-h co-culture, the corresponding luciferase activity was detected by the dual luciferase reporter gene assay kit (E1910) (Promega). The same method was utilized to verify the targeting relationship between miR-34a-3p and TRAF5.

### RNA pull down assay

Biotin-labeled miR-34a-3p WT and MUT plasmids (50 nM each) were transfected into cells severally. After 48-h transfection, the cells were incubated with a specific cell lysate (Ambion, Austin, Texas, USA) for 10 min. Then 50 mL of sample cell lysate was divided. Residual lysates were incubated with M-280 streptavidin magnetic beads precoated with RNase-free and yeast tRNA (Sigma, MO, USA) at 4℃ for 3 h. The magnetic beads were washed with cold lysate twice, low salt buffer three times, and high salt buffer once. Antagonistic miR-34a-3p probes were set as NC. Total RNA was extracted by Trizol, and DDX11-AS1 and TRAF5 expression were tested by RT-qPCR.

### Xenograft tumor in nude mice

Specific pathogen-free (SPF) grade male BALB/c nude mice aging 4–6 weeks and weighing 18–22 g were purchased from the Experimental Animal Center of Zhengzhou University (Shanghai, China). The nude mice were fed in the SPF grade animal room with constant temperature and humidity, and free to drink and eat. The experiment operation was conducted in a sterile cover. The nude mice (n = 6/group) were injected with the transfected SMMC-7721 and SK-hep1 cells.

A total of 200 μL cells (2.5 × 10^7^ cells/mL) were seeded subcutaneously in the back of nude mice. Mice were fed to observe the growth of xenografted tumors. The nude mice were euthanized on 28st days, the xenografted tumor was removed, and the tumor volume and weight were calculated. The tumor length (L) and short diameter (W) were measured, and the volume was calculated. V = (L × W^2^)/2.

### Statistical analysis

The data were statistically analyzed by SPSS 21.0 (IBM Corp., NY, USA) statistical software. The measurement data were expressed as mean ± standard deviation. The t test was used for two-group comparison in the data subjected to normal distribution. One-way analysis of variance (ANOVA) was used for comparison among multiple groups, and Tukey’s post hoc test was used for pairwise comparison. Predictors were kept if they were significant at a *P* value of 0.05 or smaller. Pearson test was used for correlation analysis.

## Results

### Up-regulated DDX11-AS1, TRAF5 and reduced miR-34a-3p exhibit in HCC tissues and cells

**Fig. 1:**
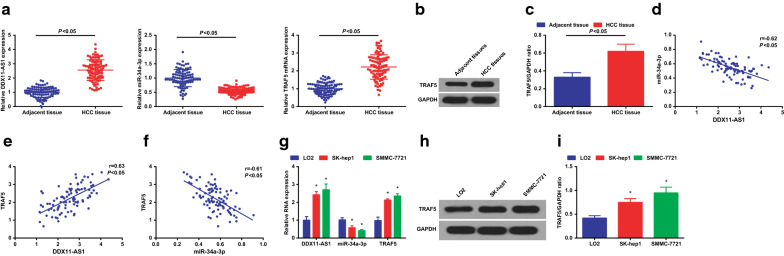
Elevated DDX11-AS1, TRAF5 and reduced miR-34a-3p are present in HCC tissues and cells. **a** DDX11-AS1, miR-34a-3p and TRAF5 expression in HCC tissues and adjacent tissues detected via RT-qPCR; **b** TRAF5 protein bands in HCC tissues and adjacent tissues; **c** TRAF5 protein expression in HCC tissues and adjacent tissues detected via western blot analysis; **d** The correlation between DDX11-AS1 and miR-34a-3p expression in HCC patients analyzed via Pearson test; **e** The correlation between DDX11-AS1 and TRAF5 expression in HCC patients analyzed via Pearson test; **f** The correlation between miR-34a-3p and TRAF5 expression in HCC patients analyzed via Pearson test; **g** DDX11-AS1, miR-34a-3p and TRAF5 expression in normal human hepatocytes LO2 and HCC cell lines SMMC-7721 and SK-hep1 detected via RT-qPCR; **h** TRAF5 protein bands in normal human hepatocytes LO2 and HCC cell lines SMMC-7721 and SK-hep1; **i** TRAF5 protein expression in normal human hepatocytes LO2 and HCC cell lines SMMC-7721 and SK-hep1 detected via western blot analysis; The measurement data were expressed as mean ± standard deviation. The t test was used for two-group comparison. One-way ANOVA was used for comparison among multiple groups, and Tukey’s post hoc test was used for pairwise comparison. Pearson test was used for correlation analysis. * vs LO2 cells, *P* < 0.05

RT-qPCR and western blot analysis were implemented to detect DDX11-AS1, miR-34a-3p and TRAF5 expression in HCC tissues and adjacent tissues. We found that (Fig. 1a–c) DDX11-AS1 and TRAF5 expression was increased while miR-34a-3p expression was decreased in HCC tissues.

Pearson test was utilized for the analysis of correlation between DDX11-AS1, miR-34a-3p and TRAF5 expression in HCC patients. The results indicated that (Fig. 1d–f), DDX11-AS1 expression had a negative correlation with miR-34a-3p expression, while a positive correlation with TRAF5 expression. In addition, miR-34a-3p expression had a negative correlation with TRAF5 expression.

DDX11-AS1, miR-34a-3p and TRAF5 expression were also detected in human normal hepatocytes LO2 and HCC cell lines SMMC-7721 and SK-hep1 (Fig. 1g–i). DDX11-AS1 and TRAF5 expression were elevated while miR-34a-3p expression was reduced in HCC cell lines SMMC-7721 and SK-hep1.

### Silenced DDX11-AS1 or up-regulated miR-34a-3p inhibits the migration, invasion, induces apoptosis of HCC cells and represses the tumor growth in nude mice

**Fig. 2:**
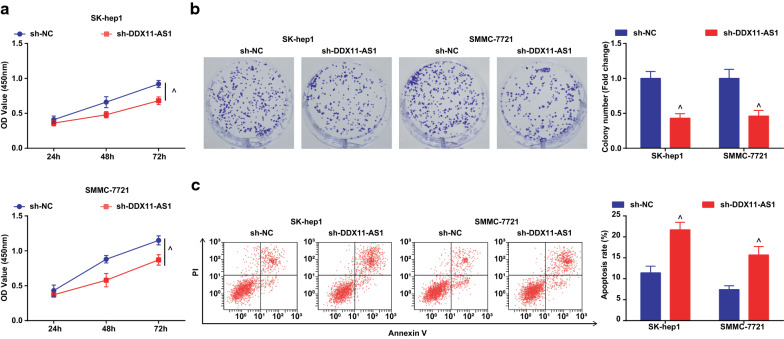
Reduced DDX11-AS1 inhibits the proliferation, promotes apoptosis of HCC cells. **a** The cell growth curve detected by CCK-8 assay; **b** The cell proliferation rate detected via colony formation assay; **c** The apoptosis rate detected via Annexin V-FITC/PI double staining; The measurement data were expressed as mean ± standard deviation. The t test was used for two-group comparison. ^ vs the sh-NC group, *P* < 0.05

**Fig. 3:**
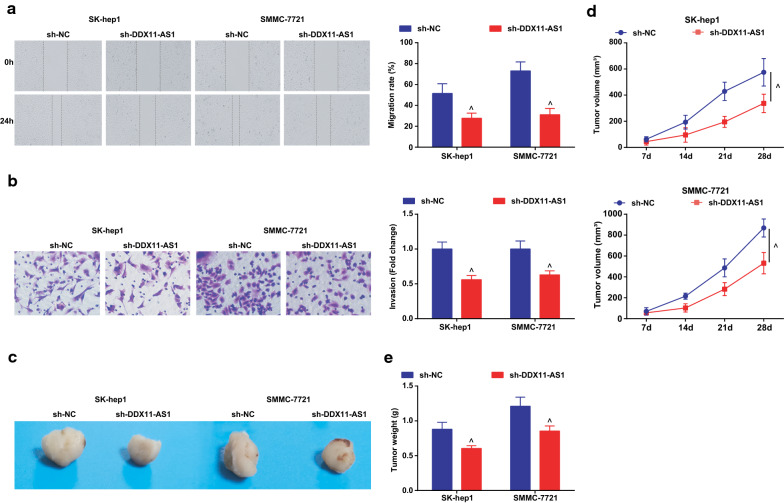
Reduced DDX11-AS1 inhibits the migration, invasion of HCC cells, promotes and represses the tumor growth in nude mice. **a** The cell migration detected by scratch test; **b** The cell invasion detected by Transwell assay; **c** Representative images of xenografted tumors in nude mice of each group; **d** Xenografted tumor volume in nude mice of each group; **e** Xenografted tumor weight in nude mice of each group; The measurement data were expressed as mean ± standard deviation. The t test was used for two-group comparison. ^ vs the sh-NC group, *P* < 0.05

**Fig. 4:**
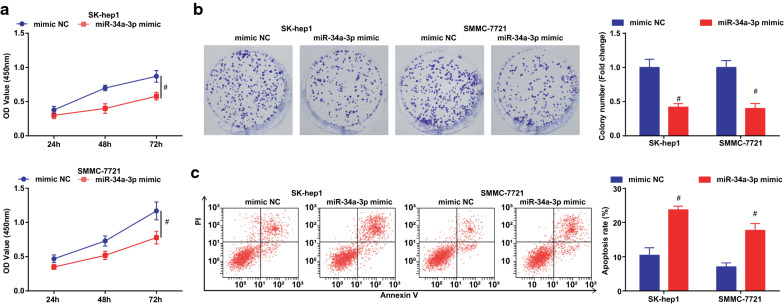
Elevated miR-34a-3p suppresses the proliferation and induces apoptosis of HCC cells.** a** The cell growth curve detected by CCK-8 assay;** b** The cell proliferation rate detected via colony formation assay;** c** The apoptosis rate detected via Annexin V-FITC/PI double staining;The measurement data were expressed as mean ± standard deviation. The t test was used for two-group comparison. # vs the mimic NC group, *P* < 0.05

**Fig. 5:**
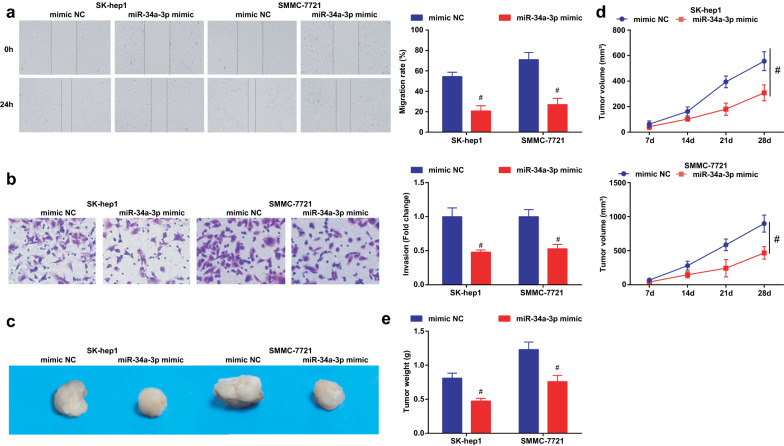
Elevated miR-34a-3p suppresses the migration, invasion of HCC cells and suppresses the tumor growth in nude mice. **a** The cell migration detected by scratch test; **b** The cell invasion detected by Transwell assay; **c** Representative images of xenografted tumors in nude mice of each group; **d** Xenografted tumor volume in nude mice of each group; **e** Xenografted tumor weight in nude mice of each group; The measurement data were expressed as mean ± standard deviation. The t test was used for two-group comparison. # vs the mimic NC group, *P* < 0.05

The effect of DDX11-AS1 silence or miR-34a-3p elevation on the malignant phenotypes of HCC cells was observed, and it was indicated that (Figs. 2a–c, 3a, b; 4a–c; 5a, b) silenced DDX11-AS1 or elevated miR-34a-3p suppressed the proliferation, migration, and invasion of HCC cells, while elevated the apoptosis rate.

The effect of DDX11-AS1 silence or miR-34a-3p elevation on the growth of xenografted tumor of HCC cells was observed (Figs. 3c–e; 5c–e). After DDX11-AS1 silence or miR-34a-3p up-regulation, the volume and weight of xenografted tumor in mice injected with HCC cells were decreased.

Ki67 and Caspase-3 mRNA levels in tumor tissues were detected by RT-qPCR, and it was found that after down-regulating DDX11-AS1 or up-regulating miR-34a-3p, Ki67 expression was decreased while Caspase-3 expression was increased in tumor tissues (Additional file 1: Figure S1A, B).

### Silenced DDX11-AS1 elevates miR-34a-3p to decrease TRAF5 expression

**Fig. 6:**
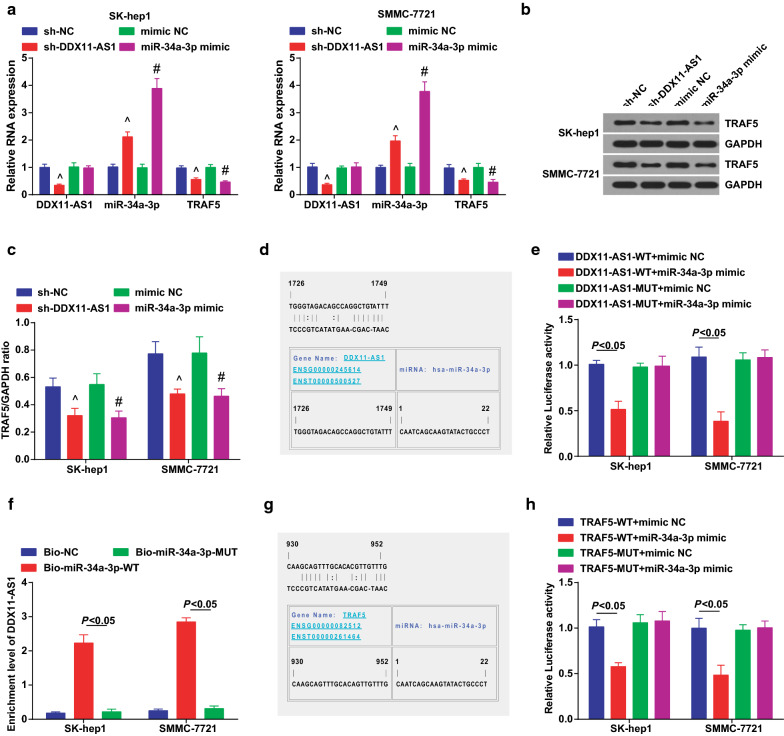
Reduced DDX11-AS1 increases miR-34a-3p to reduce TRAF5 expression. **a** DDX11-AS1, miR-34a-3p and TRAF5 expression detected by RT-qPCR; **b** TRAF5 protein bands; **c** TRAF5 protein expression detected by western blot analysis; **d** The binding sites of DDX11-AS1 and miR-34a-3p predicted via bioinformatics website https://cm.jefferson.edu/rna22/Precomputed/; **e** The regulatory relationship between DDX11-AS1 and miR-34a-3p verified via dual luciferase reporter gene assay; **f** The binding relationship between DDX11-AS1 and miR-34a-3p verified via RNA-pull down assay; **g** The targeting relationship between miR-34a-3p and TRAF5 predicted via bioinformatics website https://cm.jefferson.edu/rna22/Precomputed/; **h** The targeting relationship between miR-34a-3p and TRAF5 verified via luciferase activity assay; The measurement data were expressed as mean ± standard deviation. One-way ANOVA was used for comparison among multiple groups, and Tukey’s post hoc test was used for pairwise comparison after ANOVA analysis. ^ vs the sh-NC group, *P* < 0.05; # vs the mimic NC group, *P* < 0.05

In the above experiments, we found up-regulated DDX11-AS1 and down-regulated miR-34a-3p in HCC tissues, thus a certain correlation between DDX11-AS1 and miR-34a-3p was speculated in HCC cells. DDX11-AS1, miR-34a-3p and TRAF5 expression were detected (Fig. 6a–c), and results suggested that silenced DDX11-AS1 elevated miR-34a-3p and deceased TRAF5 expression. In addition, up-regulated miR-34a-3p reduced TRAF5 expression.

A bioinformatics software was utilized for prediction of the specific binding region between the DDX11-AS1 and miR-34a-3p (Fig. 6d). Dual luciferase reporter gene assay results indicated that (Fig. 6e) the luciferase activity of cells after co-transfection of DDX11-AS1-WT + miR-34a-3p mimic was apparently reduced, indicating a binding relationship between DDX11-AS1 and miR-34a-3p. It was revealed in RNA-pull down assay that (Fig. 6f), DDX11-AS1 expression was elevated by Bio-miR-34a-3p-WT. This indicated that Bio-miR-34a-3p-WT could promote the enrichment of DDX11-AS1, confirming that DDX11-AS1 could bind with miR-34a-3p and reduce the dissociation degree of miR-34a-3p.

TRAF5 is often used as a regulatory target gene in HCC [24]. Therefore, we speculated that miR-34a-3p targeted TRAF5 to regulate the biological functions of HCC. For verification, bioinformatics software was utilized to predict a targeting relationship between miR-34a-3p and TRAF5 (Fig. 6g). Luciferase activity test showed that (Fig. 6h), after co-transfection of TRAF5-WT with miR-34a-3p mimic, the relative luciferase activity of cells was clearly decreased. In summary, DDX11-AS1 had binding sites with miR-34a-3p, and miR-34a-3p and TRAF5 had a targeting relationship.

### DDX11-AS1 modulates the growth and metastasis of HCC cells through miR-34a-3p/TRAF5 axis

**Fig. 7:**
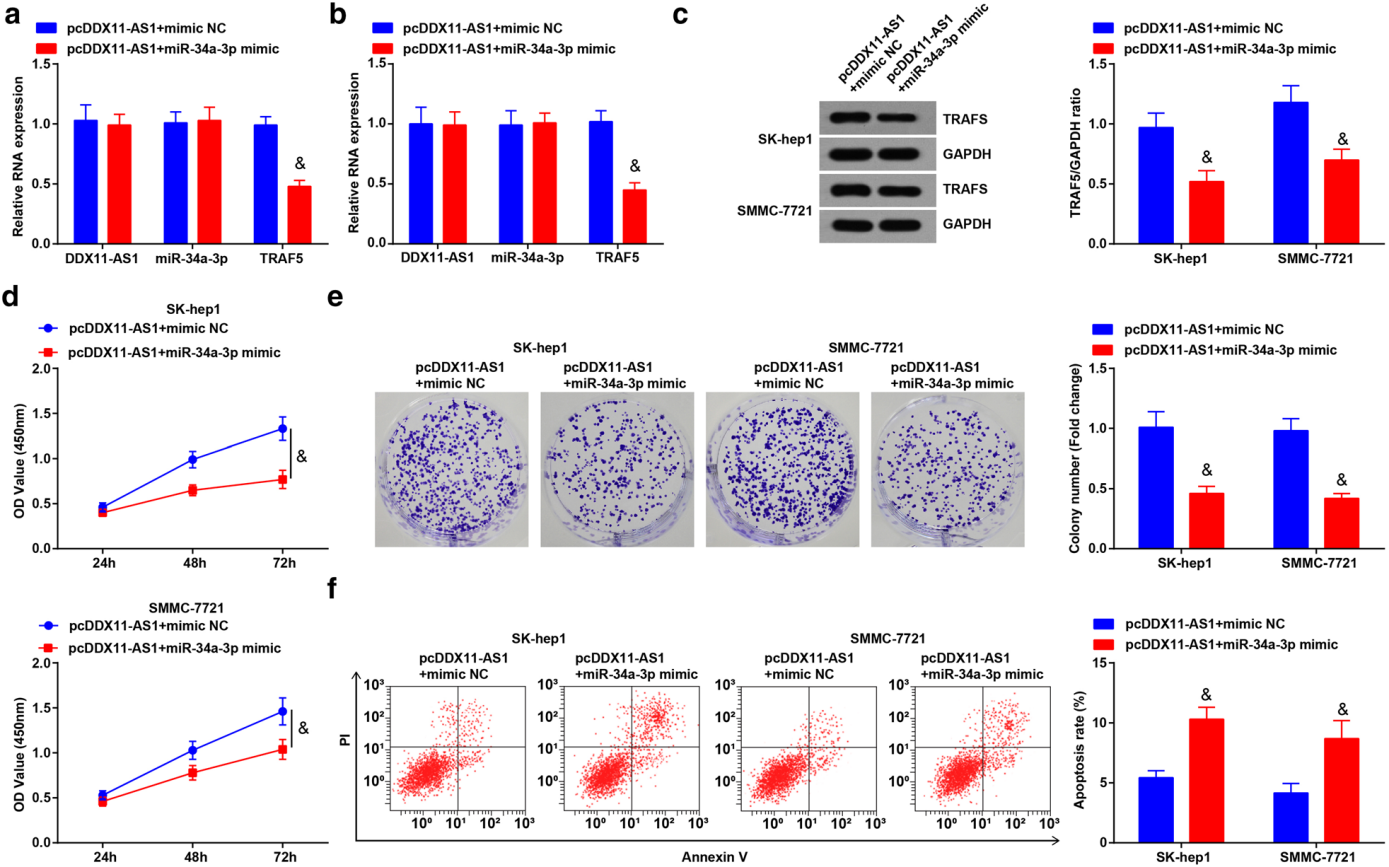
DDX11-AS1 modulates the growth and metastasis of HCC cells through miR-34a-3p/TRAF5 axis. **a**, **b** DDX11-AS1, miR-34a-3p and TRAF5 expression after co-transfection detected by RT-qPCR; **c** TRAF5 protein expression after co-transfection detected by western blot analysis; **d** The cell growth curve detected by CCK-8 assay; **e** The cell proliferation rate detected via colony formation assay; **f** The apoptosis rate detected via Annexin V-FITC/PI double staining; The measurement data were expressed as mean ± standard deviation. t test was used for comparison between two groups, One-way ANOVA for comparison among multiple groups, and Tukey’s post hoc test for pairwise comparison. & vs the pcDDX11-AS1 + mimic NC group, *P* < 0.05

**Fig. 8:**
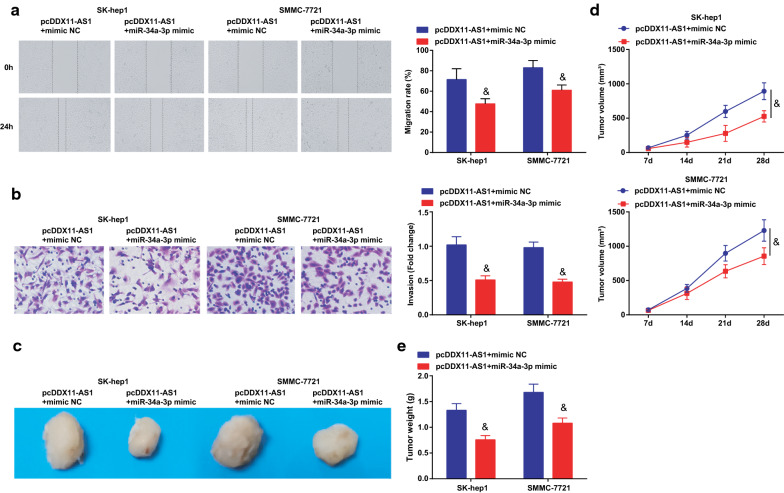
DDX11-AS1 modulates the growth and metastasis of HCC cells through miR-34a-3p/TRAF5 axis.** a** The cell migration detected by scratch test;** b** The cell invasion detected by Transwell assay;** c** Representative images of xenografted tumors in nude mice;** d** Xenografted tumor volume in nude mice of each group;** e** Xenografted tumor weight in nude mice of each group; The measurement data were expressed as mean ± standard deviation. The measurement data were expressed as mean ± standard deviation. t test was used for comparison between two groups, One-way ANOVA for comparison among multiple groups, and Tukey’s post hoc test for pairwise comparison. & vs the pcDDX11-AS1 + mimic NC group, *P* < 0.05

**Fig. 9:**
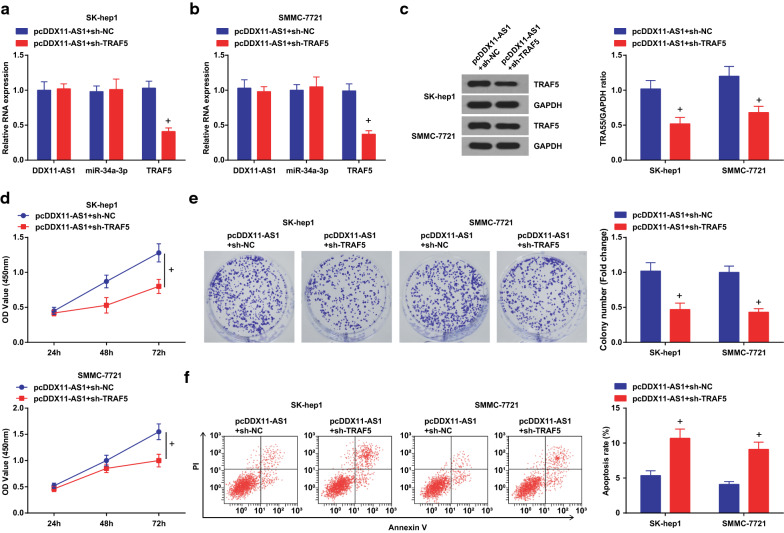
DDX11-AS1 modulates the growth and metastasis of HCC cells through miR-34a-3p/TRAF5 axis. **a**, **b** DDX11-AS1, miR-34a-3p and TRAF5 expression after co-transfection detected by RT-qPCR; **c** TRAF5 protein expression after co-transfection detected by western blot analysis; **d** The cell growth curve detected by CCK-8 assay; **e** The cell proliferation rate detected via colony formation assay; **f** The apoptosis rate detected via Annexin V-FITC/PI double staining; The measurement data were expressed as mean ± standard deviation. The measurement data were expressed as mean ± standard deviation. t test was used for comparison between two groups, One-way ANOVA for comparison among multiple groups, and Tukey’s post hoc test for pairwise comparison. + vs the pcDDX11-AS1 + sh-NC group, *P* < 0.05

**Fig. 10:**
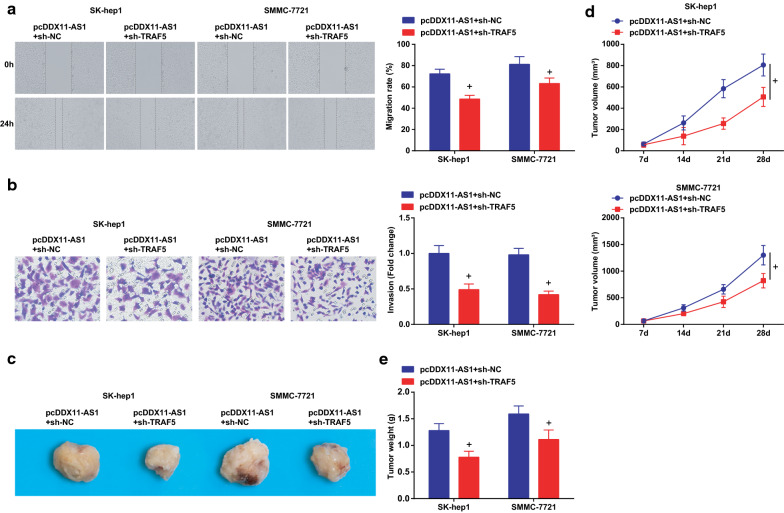
DDX11-AS1 modulates the growth and metastasis of HCC cells through miR-34a-3p/TRAF5 axis. **a** The cell migration detected by scratch test; **b** The cell invasion detected by Transwell assay; **c** Representative images of xenografted tumors in nude mice of each group; **d** Xenografted tumor volume in nude mice of each group; **e** Xenografted tumor weight in nude mice of each group; The measurement data were expressed as mean ± standard deviation. The measurement data were expressed as mean ± standard deviation. t test was used for comparison between two groups, One-way ANOVA for comparison among multiple groups, and Tukey’s post hoc test for pairwise comparison. + vs the pcDDX11-AS1 + sh-NC group, *P* < 0.05

To further explore whether DDX11-AS1 affected the growth and metastasis of HCC cells through modulating the miR-34a-3p/TRAF5 axis, RT-qPCR and Western blot analysis were tested to find reduced TRAF5 expression after co-transfection of restored DDX11-AS1 and miR-34a-3p, or that of elevated DDX11-AS1 and silenced TRAF5 (Figs. 7a-c; 9a-c). The proliferation, migration, and invasion abilities of HCC cells were suppressed, while the apoptosis rate was elevated after co-transfection of pcDDX11-AS1 and miR-34a-3p mimic, or that of pcDDX11-AS1 and sh-TRAF5 (Figs. 7d-F; 8a, b; 9d-f; 10a, b).

The effect of DDX11-AS1 with miR-34a-3p on the growth of xenografted tumor of HCC cells was observed (Figs. 8c-e; 10c-e). The volume and weight of xenografted tumor in nude mice injected with HCC cells were decreased after co-transfection of pcDDX11-AS1 and miR-34a-3p mimic, or that of pcDDX11-AS1 and sh-TRAF5. Also, it was measured that miR-34a-3p mimic or sh-TRAF5 reversed the influence of pcDDX11-AS1, thus to reduce Ki67 and increase Caspase-3 expression in tumor tissues (Additional file 1: Figure S1C, D).

## Discussion

HCC is a familiar malignancy in the world [26]. A study has manifested that DDX11-AS1 could be used as an oncogene in HCC and supplied a fresh-new therapeutic target for treatment [10]. Previous studies have manifested the relationship of miR-34a with HCC. For instance, a study has demonstrated that miR-34a is applied as a potential tumor suppressor via the regulation of telomere pathway in HCC [27]. The data of a research have suggested that suppression of miR-34a is connected with HCC progression and may perform as a suppressive role in HCC [28]. While the literature about the connection of miR-34a-3p with HCC is few. A study has validated the link of TRAF5 with HCC that down-regulated TRAF5 reverses LINC00467 elevation-mediated promotion on HCC progression [24]. To fill the blank, we aimed to investigate the regulatory mechanism of DDX11-AS1/miR-34a-3p/TRAF5 signaling axis on HCC cells.

The observation of the study was that up-regulated DDX11-AS1, TRAF5 and reduced miR-34a-3p exhibited in HCC. This is consonant with the result that DDX11-AS1 expression is greatly elevated in HCC [10]. It suits well that up-regulation of DDX11-AS1 is related to poorer survival in HCC patients [12]. A study has found that miR-34a is reduced in HCC tissues, and no heterogeneity is manifested [15]. In addition, the study has demonstrated that miR-34a serum levels are lowly expressed especially in HCC patients as compared to controls [29]. It has been evidenced that TRAF5 expression is elevated in HCC [23]. In addition, TRAF5 increase could reverse LINC00467 downregulation-mediated repression on HCC cell growth, apoptosis and metastasis [24].

In this work, we also found that silenced DDX11-AS1 elevated miR-34a-3p to inhibit TRAF5 expression. As demonstrated before, similar results were acquired. For example, DDX11-AS1 could act as a competing endogenous RNA for other miRNAs [30]. It is reported that up-regulated DDX11-AS1 competitively binds to miR-873 [31]. The most obvious result of the study was that silenced DDX11-AS1 or up-regulated miR-34a-3p depressed the growth of HCC cells. In addition, elevated miR-34a-3p reversed the promotion of DDX11-AS1 on the growth and metastasis of HCC cells. Moreover, the result was related to the suppression of TRAF5. This finding is also reported by Tian et al*.* that the decrease of DDX11-AS1 represses the proliferation, migration, and invasion of colorectal cancer cells, and induces apoptosis [31]. This also accords with our earlier observations, which shows that knocking down DDX11-AS1 decreases TOP2A expression and represses tumor growth of esophageal cancer [7]. These results reflect those of Werner et al*.* who also find that overexpressed miR-34a-3p down-regulates the proliferation of meningioma cells and decreased apoptosis is found after repression of miR-34a-3p [32]. In addition, SiHa cell transfection of miR-34a-3p exhibits a cell proliferation suppression in cervical cancer and miR-34a-3p could also decline cell motility [33]. Furthermore, TRAF5 with other elements is able to suppress the invasion and migration abilities of prostate cancer cells [34]. The study has manifested that TRAF with other factors represses the proliferation, migration, and invasion in CRC [35]. The aforementioned evidences confirm that silenced DDX11-AS1 or up-regulated miR-34a-3p contributes to suppressed growth of different types of cancers through downregulating TRAF5.

In summary, the study concludes that silenced DDX11-AS1 or up-regulated miR-34a-3p inhibits the growth of HCC cells and represses the tumor growth in nude mice via repression of TRAF5. This finding has important implications for exploring the pathogenesis of HCC. The results of this paper can be further verified to identify the binding relationship between DDX11-AS1 and miR-34a-3p and the targeting relationship between miR-34a-3p and TRAF5 in HCC in the future.

## Supplementary Information

**Additional file 1: Figure S1.** The effect of DDX11-AS1/miR-34a-3p/TRAF5 on the malignant phenotype of xenografts. A–D. RT-qPCR detection of Ki67 and Caspase-3 mRNA levels in tumor tissues. The measurement data were expressed as mean ± standard deviation. The measurement data were expressed as mean ± standard deviation. t test was used for comparison between two groups, One-way ANOVA for comparison among multiple groups, and Tukey's post hoc test for pairwise comparison. ^ vs the sh-NC group, *P* < 0.05; # vs the mimic NC group, *P* < 0.05; & vs the pcDDX11-AS1 + mimic NC group, *P* < 0.05; + vs the pcDDX11-AS1 + sh-NC group, *P* < 0.05.

## Data Availability

Not applicable.

## References

[1] Zhang J (2020). The threshold of alpha-fetoprotein (AFP) for the diagnosis of hepatocellular carcinoma: A systematic review and meta-analysis. PLoS ONE.

[2] Zhao S (2020). Evodiamine inhibits proliferation and promotes apoptosis of hepatocellular carcinoma cells via the Hippo-Yes-Associated Protein signaling pathway. Life Sci.

[3] Zhang GP (2020). Kinesin family member 2C aggravates the progression of hepatocellular carcinoma and interacts with competing endogenous RNA. J Cell Biochem.

[4] Graf D (2014). Multimodal treatment of hepatocellular carcinoma. Eur J Intern Med.

[5] Tsilimigras DI (2020). Hepatocellular carcinoma tumour burden score to stratify prognosis after resection. Br J Surg.

[6] Uthaya Kumar DB, Williams A (2020). Long non-coding RNAs in immune regulation and their potential as therapeutic targets. Int Immunopharmacol.

[7] Zhang S (2019). The resistance of esophageal cancer cells to paclitaxel can be reduced by the knockdown of long noncoding RNA DDX11-AS1 through TAF1/TOP2A inhibition. Am J Cancer Res.

[8] Shi M (2017). DDX11-AS1 as potential therapy targets for human hepatocellular carcinoma. Oncotarget.

[9] Li Q (2020). DDX11-AS1exacerbates bladder cancer progression by enhancing CDK6 expression via suppressing miR-499b-5p. Biomed Pharmacother.

[10] Li Y (2019). Long noncoding RNA DDX11-AS1 epigenetically represses LATS2 by interacting with EZH2 and DNMT1 in hepatocellular carcinoma. Biochem Biophys Res Commun.

[11] Wan T (2021). LncRNA DDX11-AS1 accelerates hepatocellular carcinoma progression via the miR-195-5p/MACC1 pathway. Ann Hepatol.

[12] Liao HT (2018). Identification of the aberrantly expressed LncRNAs in hepatocellular carcinoma: a bioinformatics analysis based on RNA-sequencing. Sci Rep.

[13] Escate R (2020). High miR-133a levels in the circulation anticipates presentation of clinical events in familial hypercholesterolemia patients. Cardiovasc Res.

[14] Soliman B (2018). Bioinformatics functional analysis of let-7a, miR-34a, and miR-199a/b reveals novel insights into immune system pathways and cancer hallmarks for hepatocellular carcinoma. Tumour Biol.

[15] Ren FH (2018). Analysis of microarrays of miR-34a and its identification of prospective target gene signature in hepatocellular carcinoma. BMC Cancer.

[16] Jiang X (2020). LncRNA OIP5-AS1 upregulates snail expression by sponging miR-34a to promote ovarian carcinoma cell invasion and migration. Biol Res.

[17] Ma E (2020). LINC01006 facilitates cell proliferation, migration and invasion in prostate cancer through targeting miR-34a-5p to up-regulate DAAM1. Cancer Cell Int.

[18] Feili X (2018). MicroRNA-34a-5p inhibits liver fibrosis by regulating TGF-beta1/Smad3 pathway in hepatic stellate cells. Cell Biol Int.

[19] Zhu S (2018). Genetic Alterations of TRAF Proteins in Human Cancers. Front Immunol.

[20] Li J (2020). The relationship between TRAF6 and tumors. Cancer Cell Int.

[21] Song P (2019). The regulatory protein GADD34 inhibits TRAIL-induced apoptosis via TRAF6/ERK-dependent stabilization of myeloid cell leukemia 1 in liver cancer cells. J Biol Chem.

[22] Teng YL (2019). Overexpression of miRNA-410-3p protects hypoxia-induced cardiomyocyte injury via targeting TRAF5. Eur Rev Med Pharmacol Sci.

[23] Jiang W (2019). LINC00467 promotes cell proliferation and metastasis by binding with IGF2BP3 to enhance the mRNA stability of TRAF5 in hepatocellular carcinoma. J Gene Med.

[24] Ling ZA (2018). LncRNA NEAT1 promotes deterioration of hepatocellular carcinoma based on in vitro experiments, data mining, and RT-qPCR analysis. Cell Physiol Biochem.

[25] Fang Y (2017). TFE3 regulates renal adenocarcinoma cell proliferation via activation of the mTOR pathway. Mol Med Rep.

[26] Irie T (2020). Balloon-occluded trans-arterial chemo-embolization technique with repeated alternate infusion of cisplatin solution and sparse gelatin slurry (RAIB-TACE) for large hepatocellular carcinoma nodules more than 7 cm in diameter. Biomed Res Int.

[27] Xu X (2015). miR-34a induces cellular senescence via modulation of telomerase activity in human hepatocellular carcinoma by targeting FoxM1/c-Myc pathway. Oncotarget.

[28] Tian YW (2017). Decreased levels of miR-34a and miR-217 act as predictor biomarkers of aggressive progression and poor prognosis in hepatocellular carcinoma. Minerva Med.

[29] Shehata RH (2017). Deregulation of miR-34a and its chaperon Hsp70 in hepatitis C virus-induced liver cirrhosis and hepatocellular carcinoma patients. Asian Pac J Cancer Prev.

[30] Ren Z (2020). Long non-coding RNA DDX11-AS1 facilitates gastric cancer progression by regulating miR-873-5p/SPC18 axis. Artif Cells Nanomed Biotechnol.

[31] Tian JB, Cao L, Dong GL (2019). Long noncoding RNA DDX11-AS1 induced by YY1 accelerates colorectal cancer progression through targeting miR-873/CLDN7 axis. Eur Rev Med Pharmacol Sci.

[32] Werner TV (2017). MiR-34a-3p alters proliferation and apoptosis of meningioma cells in vitro and is directly targeting SMAD4, FRAT1 and BCL2. Aging (Albany NY).

[33] Cordova-Rivas S (2019). 5p and 3p strands of miR-34 family members have differential effects in cell proliferation, migration, and invasion in cervical cancer cells. Int J Mol Sci.

[34] Peng P (2019). Decreased miR-218-5p Levels as a Serum Biomarker in Bone Metastasis of Prostate Cancer. Oncol Res Treat.

[35] Liang Z (2019). MiR-141-3p inhibits cell proliferation, migration and invasion by targeting TRAF5 in colorectal cancer. Biochem Biophys Res Commun.

