# Correction to: Silenced lncRNA DDX11-AS1 or up-regulated microRNA-34a-3p inhibits malignant phenotypes of hepatocellular carcinoma cells via suppression of TRAF5

**DOI:** 10.1186/s12935-021-02360-6

**Published:** 2021-12-09

**Authors:** Gangqiang Ding, Yanli Zeng, Dongqiang Yang, Can Zhang, Chongshan Mao, Erhui Xiao, Yi Kang, Jia Shang

**Affiliations:** Department of Infectious Diseases, Henan Key Laboratory for Liver Disease, Henan Provincial People’s Hospital, People’s Hospital of Zhengzhou University, No. 7 Weiwu Road, Zhengzhou, 450003 Henan China

## Correction to: Cancer Cell Int (2021) 21:179 10.1186/s12935-021-01847-6

In this article [1], Supplement Figure 1 was misplaced. The correct supplement figure 1 is published in this correction (Additional file 1: Figure S1).

## Supplementary Information

**Additional file 1: Figure S1.** The effect of DDX11-AS1/miR-34a-3p/TRAF5 on the malignant phenotype of xenografts. A–D. RT-qPCR detection of Ki67 and Caspase-3 mRNA levels in tumor tissues. The measurement data were expressed as mean ± standard deviation. t test was used for comparison between two groups, One-way ANOVA for comparison among multiple groups, and Tukey’s post hoc test for pairwise comparison. ^vs the sh-NC group, *P* < 0.05; # vs the mimic NC group, *P* < 0.05; # vs the pcDDX11-AS1 + mimic NC group, *P* < 0.05; + vs the pcDDX11-AS1 + sh-NC group, *P* < 0.05.
