# Supplementary material for: Silenced lncRNA DDX11-AS1 or up-regulated microRNA-34a-3p inhibits malignant phenotypes of hepatocellular carcinoma cells via suppression of TRAF5
Source: Cancer Cell Int. 2021 Mar 22;21:179. doi: 10.1186/s12935-021-01847-6 (PMC7983398; doi:10.1186/s12935-021-01847-6)
Supplement: Supplementary file 1 — Additional file 1: Figure S1. The effect of DDX11-AS1/miR-34a-3p/TRAF5 on the malignant phenotype of xenografts. A–D. RT-qPCR detection of Ki67 and Caspase-3 mRNA levels in tumor tissues. The measurement data were expressed as mean ± standard deviation. The measurement data were expressed as mean ± standard deviation. t test was used for comparison between two groups, One-way ANOVA for comparison among multiple groups, and Tukey's post hoc test for pairwise comparison. ^ vs the sh-NC group, P < 0.05; # vs the mimic NC group, P < 0.05; & vs the pcDDX11-AS1 + mimic NC group, P < 0.05; + vs the pcDDX11-AS1 + sh-NC group, P < 0.05. [file 12935_2021_1847_MOESM1_ESM.docx]

Figure 1B


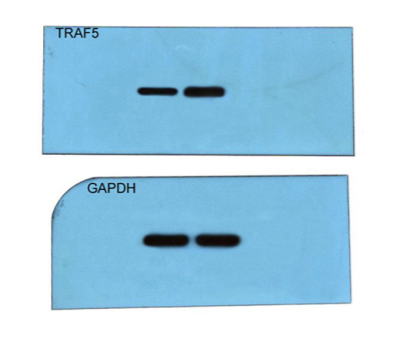


Original images of blots for Figure 1B

Figure 1H


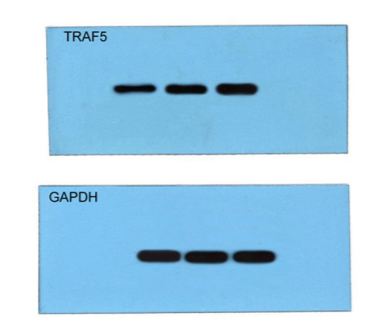


Original images of blots for Figure 1H

Figure 6B


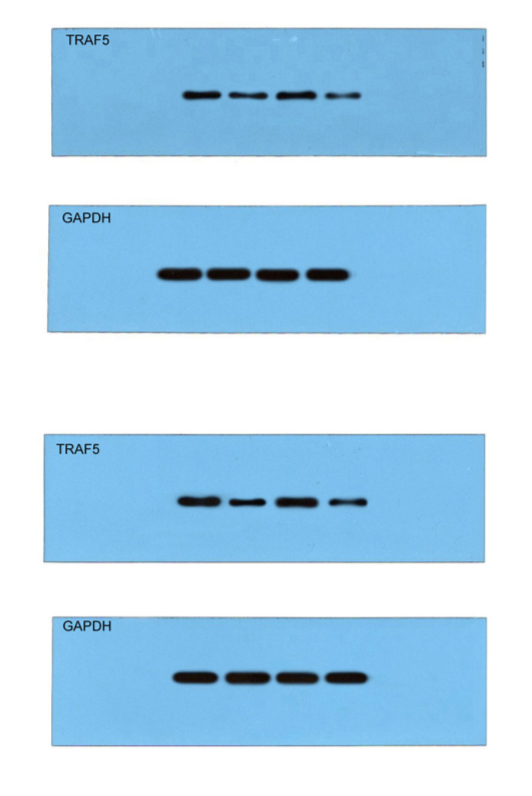


Original images of blots for Figure 6B

Figure 7C


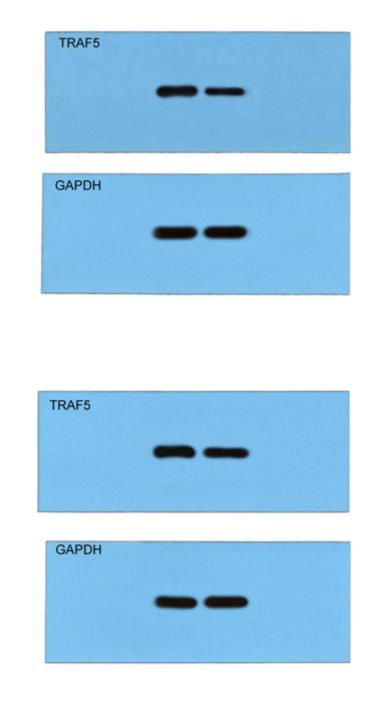


Original images of blots for Figure 7C

Figure 9C


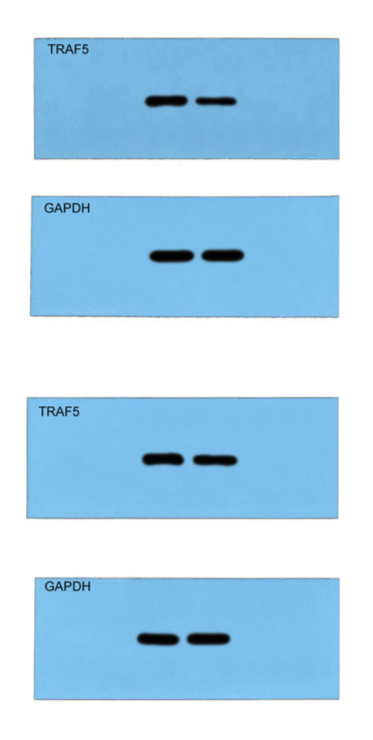


Original images of blots for Figure 9C
