# Supplementary figures and images for: Correction to: Silenced lncRNA DDX11-AS1 or up-regulated microRNA-34a-3p inhibits malignant phenotypes of hepatocellular carcinoma cells via suppression of TRAF5
Source: Cancer Cell Int. 2021 Dec 9;21:659. doi: 10.1186/s12935-021-02360-6 (PMC8662869; doi:10.1186/s12935-021-02360-6)

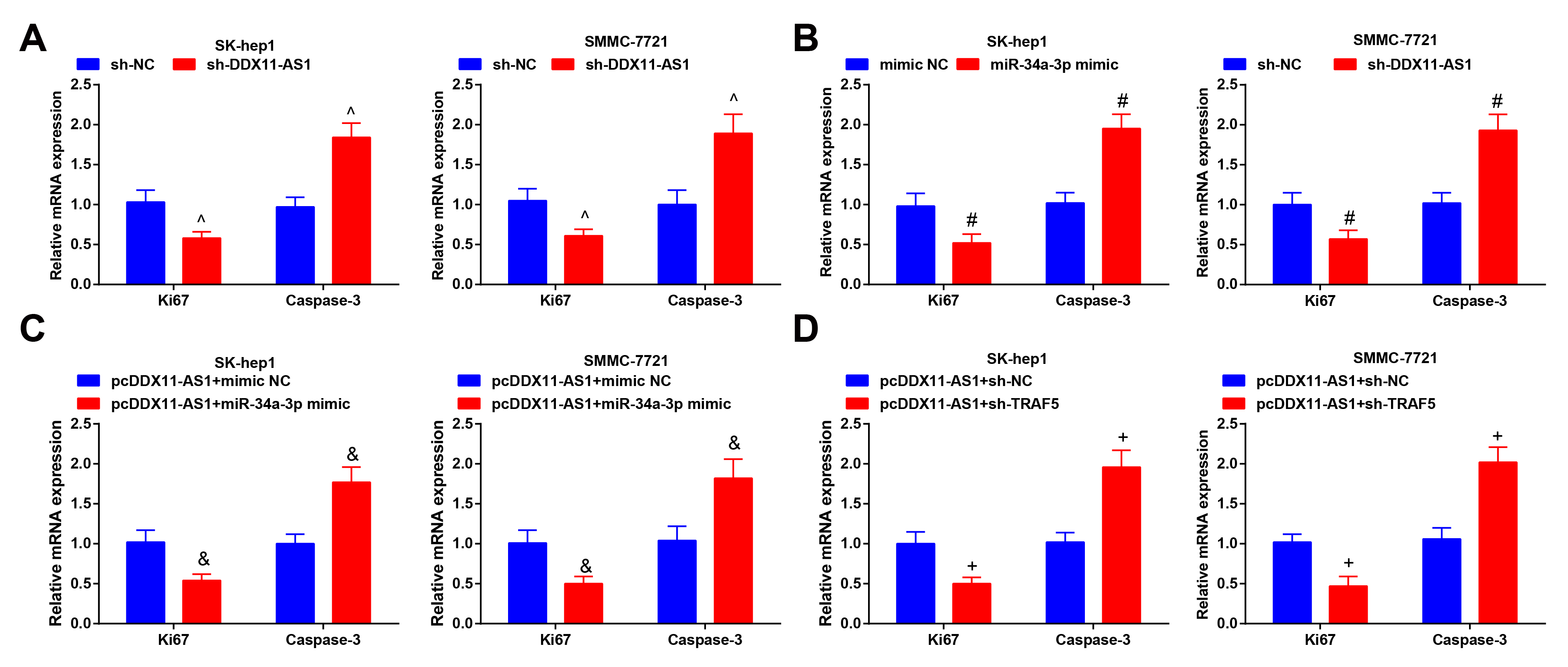

Supplement: Supplementary file 1 — Additional file 1: Figure S1. The effect of DDX11-AS1/miR-34a-3p/TRAF5 on the malignant phenotype of xenografts. A–D. RT-qPCR detection of Ki67 and Caspase-3 mRNA levels in tumor tissues. The measurement data were expressed as mean ± standard deviation. t test was used for comparison between two groups, One-way ANOVA for comparison among multiple groups, and Tukey’s post hoc test for pairwise comparison. ^vs the sh-NC group, P < 0.05; # vs the mimic NC group, P < 0.05; # vs the pcDDX11-AS1 + mimic NC group, P < 0.05; + vs the pcDDX11-AS1 + sh-NC group, P < 0.05. [file 12935_2021_2360_MOESM1_ESM.jpg]
